# Intercellular Transfer of Mitochondria between Senescent Cells through Cytoskeleton-Supported Intercellular Bridges Requires mTOR and CDC42 Signalling

**DOI:** 10.1155/2021/6697861

**Published:** 2021-07-31

**Authors:** Hannah E. Walters, Lynne S. Cox

**Affiliations:** ^1^Department of Biochemistry, University of Oxford, South Parks Road, Oxford OX1 3QU, UK; ^2^Technische Universität Dresden, CRTD/Center for Regenerative Therapies Dresden, Dresden, Germany

## Abstract

Cellular senescence is a state of irreversible cell proliferation arrest induced by various stressors including telomere attrition, DNA damage, and oncogene induction. While beneficial as an acute response to stress, the accumulation of senescent cells with increasing age is thought to contribute adversely to the development of cancer and a number of other age-related diseases, including neurodegenerative diseases for which there are currently no effective disease-modifying therapies. Non-cell-autonomous effects of senescent cells have been suggested to arise through the SASP, a wide variety of proinflammatory cytokines, chemokines, and exosomes secreted by senescent cells. Here, we report an additional means of cell communication utilised by senescent cells via large numbers of membrane-bound intercellular bridges—or tunnelling nanotubes (TNTs)—containing the cytoskeletal components actin and tubulin, which form direct physical connections between cells. We observe the presence of mitochondria in these TNTs and show organelle transfer through the TNTs to adjacent cells. While transport of individual mitochondria along single TNTs appears by time-lapse studies to be unidirectional, we show by differentially labelled co-culture experiments that organelle transfer through TNTs can occur between different cells of equivalent cell age, but that senescent cells, rather than proliferating cells, appear to be predominant mitochondrial donors. Using small molecule inhibitors, we demonstrate that senescent cell TNTs are dependent on signalling through the mTOR pathway, which we further show is mediated at least in part through the downstream actin-cytoskeleton regulatory factor CDC42. These findings have significant implications for the development of senomodifying therapies, as they highlight the need to account for local direct cell-cell contacts as well as the SASP in order to treat cancer and diseases of ageing in which senescence is a key factor.

## 1. Introduction

Intercellular communication is crucial in regulating cellular function, for example, in response to environmental or intracellular stress. Such interplay has historically been thought to be coordinated through the secretion of soluble factors including chemokines, cytokines, growth factors, and hormones, and their recognition by cell surface receptors or through the secretion and internalisation of extracellular vesicles. However, recent evidence suggests that an alternative form of cell-cell communication can be mediated through intercellular membrane connections. Such connections, termed tunnelling nanotubes (TNTs) or intercellular bridges, have been characterised as long, fragile, open-ended and transient protrusions which mediate membrane continuity between connected cells. Since their initial description [1], nanotubes have been observed connecting cells of the same or different cell types, with a particular prevalence detected in immune cells including macrophages, monocytes and NK cells [2]. The cargo shuttled within nanotubes includes nutrients, sterols, plasma membrane components, signalling molecules, proteins, RNA species and ions that passively diffuse between connected cells, alongside larger cargos such as whole organelles or protein complexes that require transport by myosin motors [3]. Nanotubes may play important physiological functions, for example, in osteoclast differentiation [4], and have been observed *in vivo*, both in murine myeloid cells in the cornea [5] and in human malignant pleural mesothelioma [6].

Intercellular bridges can form either by protrusion elongation, where one cell extends filopodia-like protrusions which subsequently connect with a second nearby cell, or by cell dislodgement, where two cells which are initially close move apart, leaving behind a long, thin membrane connection [2]. Both result in the formation of nanotubes with diameters ranging from 50 to 1500 nm, spanning tens to hundreds of microns between connected cells. Actin filaments (together with myosin) are present in “thin” TNTs, while “thick” TNTs additionally contain microtubules, raising the possibility that cytoskeletal composition could control TNT cargo transport through size exclusion [7]. Given the universal requirement for F-actin in bridge structures, it is unsurprising that regulators of actin cytoskeletal dynamics including Rac1, CDC42, and their respective downstream effectors WAVE and WASP are also implicated in bridge formation, as well as the myosin motor protein Myo10 [8]. CDC42 expression, which also regulates filopodia formation, appears important for both bridge elongation and intercellular cargo transport [9].

Intercellular communication through TNTs may represent a cellular response to stress, potentially allowing rescue through transfer of functional components from undamaged cells nearby. Consistent with this, both oxidative stress and DNA damage are associated with increased TNT formation [10], in a manner dependent on p53 [11]. Stressed cells appear to “reach out” to unstressed cells [11], and transfer of mitochondria from healthy neighbouring cells to stressed cells via TNTs has been speculated to serve as a rescue mechanism for stress tolerance. For example, intercellular mitochondrial transport allows survival of cancer cells experiencing loss of mitochondrial functionality [12]. As well as providing rescue from metabolic failure or mitochondrial dysfunction, mitochondrial transfer via TNTs has even been reported to result in cellular reprogramming [13]. Nanotube formation may also be involved in the induction of apoptosis, as the death signal Fas ligand has been noted to be shuttled via TNTs in T lymphocytes to induce cell death in target cells [14].

Stress is therefore associated with TNT formation, but cellular stress is also a known driver of cell senescence (reviewed by [15]). It is hence noteworthy that intercellular membrane connections have been observed to increase upon induction of cell senescence, with transfer of cytoplasmic proteins preferentially from senescent cells to natural killer (NK) cells in co-culture [16], resulting in increased NK cell activation and cytotoxicity. Proteomic analysis of the transferred cargo showed transfer of proteins implicated in actin reorganisation. In particular, CDC42 was reported to be substantially upregulated and highly active in senescent versus proliferating fibroblasts, while its inhibition substantially reduced protein transfer and NK cytotoxicity [16].

Here, we set out to investigate the composition and role of intercellular bridges in cell senescence and whether the bridges are capable of supporting transfer of organelles between cells. We report a high prevalence of membrane-bound TNTs formed by senescent cells, containing both actin and tubulin. We further show that mitochondria can be transferred through these bridges and that mTOR signalling and CDC42-mediated actin organisation pathways are critical for organelle transfer through tunnelling nanotubes in senescent cells. These findings highlight a potential target for new therapies directed against senescent cells.

## 2. Materials and Methods

### 2.1. Cell Culture

HF043 neonatal foreskin fibroblasts (Dundee C l products) were verified to be primary diploid human fibroblasts, uncontaminated with any known lab cell line, by short tandem repeat analysis (Porton Down, UK). IMR90 ER:RAS fibroblasts (16-week female foetal lung fibroblasts) were a gift from Prof. Peter Adams (University of Glasgow, UK, and Sanford Burnham Prebys Medical Discovery Institute, La Jolla, USA). Cells were cultured in DMEM (D5796, Gibco) supplemented with 10% heat-inactivated FCS (Gibco) for HF043 or 1 mM sodium pyruvate (Gibco) and 20% FCS for IMR90 ER:RAS fibroblasts. (Media were not supplemented with antibiotics.) Cells were subcultured once they reached ~70% confluence as assessed using a digital EVOS microscope (Thermo Fisher), by washing in PBS (Sigma), 3-5 minute incubation with TrypLE Express trypsin (Thermo Fisher), and dilution and gentle trituration in complete media. Cell viability and number were assessed using a T4 Cellometer (Nexcelom), from which population doublings were subsequently calculated as PD = (log[number harvested/number seeded])/log(2). Cells were seeded at 4-8 × 10^3^ cells/cm^2^ in filter-capped flasks or multiwell plates (Greiner) and incubated with complete media in a humidified incubator at 37°C in 5% CO_2_ and 20% O_2_. Cells were regularly inspected by phase contrast microscopy for cell health and tested for mycoplasma contamination by PCR according to the method of Uphoff and Drexler [17, 18].

### 2.2. Drug Treatments

All drugs used were reconstituted and stored as directed by the supplier (etoposide, 4-hydroxytamoxifen (4-OHT), mitomycin C, and CASIN—all from Sigma-Aldrich; AZD8055—Selleckchem). For routine drug treatment, cells were seeded in complete media and allowed to bed down overnight, before media were aspirated and replaced with drug-supplemented complete media. For drug treatment in co-culture assays, cells were seeded directly into drug-supplemented complete media. Optimum concentrations and dosing periods for induction of senescence without cell killing were selected according to previously published experiments [19–22].

### 2.3. Induction and Assessment of Senescence

#### 2.3.1. Replicative Senescence (RS)

The primary human fibroblast line HF043 was grown in continuous culture until replicative exhaustion. Cells were determined to be replicatively senescent when populations fulfilled each of the following criteria: failure to increase the cell number within >2 weeks, a cumulative population doubling number of >85, and positive SA-*β*-gal staining according to the manufacturer's instructions (Cell Signaling Technology #9860S). The secretion of IL-6, a canonical SASP factor, and upregulation of p21^CDKN1^ (see Supplementary Figures S1, S2, and S3) were assessed as additional readouts of senescence (see [23] for western blotting conditions and [20] for ELISA protocol).

#### 2.3.2. DNA Damage-Induced Senescence (DDIS)

Cells were treated for 7 days with 20 *μ*M etoposide or 10 nM mitomycin C and verified as senescent by SA-*β*-gal staining and morphological assessment, as well as upregulation of p21^CDKN1^ (Supplementary Figures S2 and S3). Cells at low CPD (<50) were always used for DNA damage-induced senescence to avoid confounding effects of replicative senescence.

#### 2.3.3. Oncogene-Induced Senescence (OIS)

IMR90 ER:RAS cells were incubated with 1 *μ*M 4-OHT for 7 days and assessed for senescence induction by SA-*β*-gal staining, morphological assessment (Supplementary Figure S2), and failure to re-proliferate.

### 2.4. Fluorescence Staining

For live imaging of mitochondria, cells were incubated for 30 minutes with MitoTracker Green FM or MitoTracker Red (1 : 1000 *v*/*v* dilution of 1 mM stock) according to the manufacturer's instructions (Invitrogen Molecular Probes), in complete media at 37°C in the dark. Media were replaced before imaging to avoid fluorescent flare. Alternatively, cells were incubated overnight with a GFP-BacMam probe for mitochondria (GFP fused to the leader sequence of E1 *α* pyruvate dehydrogenase) according to the manufacturer's instructions (CellLight, Invitrogen). For co-culture experiments, cells were stained with the appropriate label, washed 3x in PBS, and harvested by trypsin treatment (as above) before reseeding at 1 : 1 ratios. To assess potential confounding dye leakage, conditioned media were harvested at 24 h from stained cells and incubated for ≥24 h with control unstained cells.

For analysis of fixed samples, mitochondria were stained for mitochondrial-specific TFAM and analysed by immunofluorescence. Briefly, cells were washed in PBS, fixed in 3.7% formaldehyde (10 minutes, RT), washed twice in PBS, blocked in 5% donkey serum (Dako) in PBS, and incubated with the primary antibody (*α*-TFAM (mouse) Ab, Abnova B01P) diluted 1 : 200 in PBS containing 0.3% Triton X-100 *v*/*v* and 1% BSA *w*/*v*, at 4°C overnight in a humidified chamber. Cells were then washed twice in PBS and incubated with the secondary antibody (Alexa Fluor 488 *α*-mouse IgG (donkey), Invitrogen A-2102); 2 hours, RT in the dark, and 1 : 500 in a dilution buffer as for the primary antibody. Cells were washed twice in PBS and DNA-counterstained before imaging.

For F-actin staining, cells were washed in PBS, fixed in 3.7% *v*/*v* formaldehyde in PBS (10 minutes, RT) before washing in PBS, and then incubated for 40 minutes with FITC-phalloidin (6.6 nM in PBS, Molecular Probes, Thermo Fisher). Cells were then washed in PBS and imaged.

For tubulin staining, cells were incubated with Tubulin Tracker Green diluted in complete media according to the manufacturer's instructions (30 minutes, 37°C, Thermo Fisher), media were replaced, and cells were imaged live.

Plasma membranes were labelled by incubating cells with 1 : 250 wheat germ agglutinin (WGA, *v*/*v*) conjugated with the fluorophore FITC or rhodamine (Vector Labs), either live in complete media (immediately after staining to avoid WGA-induced cytotoxicity) or after 3.7% formaldehyde fixation in PBS (without permeabilisation). Our optimisation studies comparing live imaging with fixed cells suggested that while fixation improved the sharpness of cell staining, it did so without disrupting the intercellular bridge structures (data not shown).

DNA was stained using NucBlue Live (Hoechst 33342) according to the manufacturer's instructions (20 minutes, 1 drop/ml, and RT, Thermo Fisher) or using Hoechst 33342 within mounting dye (VECTASHIELD, Vector Labs) for fixed cells, with 1 drop of the mounting medium used for coverslip mounting.

### 2.5. Microscopy

Phase contrast microscopy was performed using a digital EVOS Core microscope (Thermo Fisher), with scale bars added to images using a graticule. Phase contrast microscopy was used for routine assessment of cells to determine % confluence before subculturing, as well as for morphological analysis. Fluorescence microscopy was performed using a ZOE fluorescent cell imager (Bio-Rad), with scale bars added automatically. For time-lapse imaging, the field of view was locked and images taken at 5-10 min intervals, timed according to a stopwatch. Within individual experiments, optical gain was fixed at the outset of image acquisition to ensure image-to-image comparability. Between experiments, brightness and (where stated) sharpness were normalised across different samples within PowerPoint. Where necessary, Fiji software was used to enhance colour discrimination.

### 2.6. Statistical Analysis

GraphPad Prism 9 statistical analysis package was used to perform statistical tests. For comparisons between >2 samples, ANOVAs (analysis of variance) were performed, with Tukey's tests to compare the means of each sample. All graphs show mean values with standard deviations (as error bars), calculated from *n* ≥ 3 independent experiments or from technical triplicates from representative experiments of *n* ≥ 3 unless otherwise stated.

## 3. Results

### 3.1. Intercellular Bridges Connect Senescent Cells

**Figure 1:**
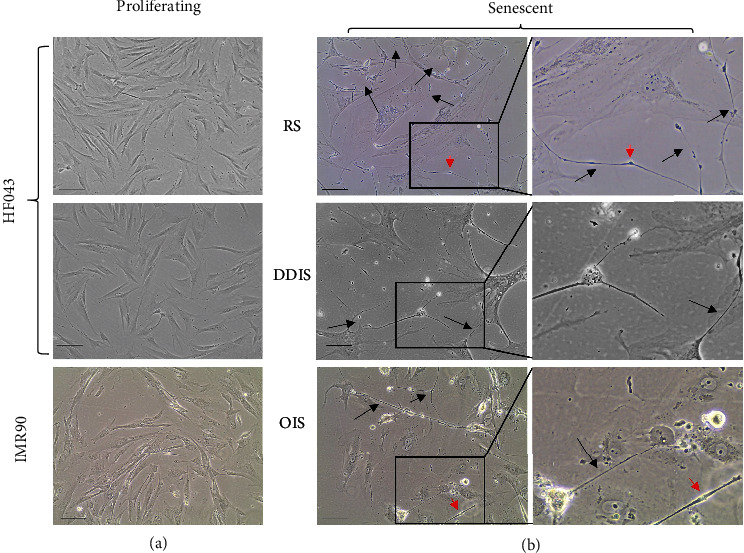
Senescent cells are connected by thin intercellular contacts *in vitro*. (a) Phase contrast images of proliferating HF043 fibroblasts at low cumulative population doubling (CPD < 40) and vehicle control proliferating IMR90 ER:RAS fibroblasts. (b) HF043 fibroblasts that have undergone replicative senescence (RS, CPD > 90) or DNA damage-induced senescence (DDIS, following 7-day 20 *μΜ* etoposide treatment), together with oncogene-induced senescent (OIS, 7-day 4-OHT) ER:RAS fibroblasts (right panels are enlarged inset regions from left panels). Black arrows indicate examples of intercellular bridges, and red arrowheads indicate bulky protrusions within the bridges. Representative data shown of *n* > 3 experiments. Scale bar 100 *μ*m.

To examine whether intercellular bridges are associated with cell senescence, we induced replicative senescence (RS) in primary human skin fibroblasts (HF043) by longitudinal continuous cell culture (serial passaging) or induced DNA damage-induced senescence (DDIS) by treating cells with etoposide for 7 days (see Materials and Methods). In parallel, we treated IMR90 ER:RAS lung fibroblasts with tamoxifen (4-OHT) to trigger oncogene-induced senescence (OIS) and compared these with proliferating control cells. As expected, the proliferating cells showed characteristic spindle-like morphology, relatively small size and were mononuclear (Figure 1(a)), while the senescent cells by contrast were greatly enlarged, contained granular inclusions, and were often multinucleated with prominent nucleoli, and cell margins were poorly defined under phase contrast optics (Figure 1(b)).

We observed by phase contrast microscopy a substantial number of long, thin structures of considerable length (tens to hundreds of microns) directly connecting nearby cells within senescent populations of both skin (HF043) and lung (IMR90) fibroblasts, irrespective of the mode of senescence induction (Figure 1(b), bridges indicated by black arrows). The bridge-like structures that we observe in the senescent cell populations were infrequent within proliferating control populations (Figure 1(a)), in agreement with previous data, suggesting that nanotube formation occurs in response to cellular stress [16], since senescence is a stress response. We further noted a number of swellings at various points along the nanotubes (red arrows, Figure 1(b)). These appear similar to protrusions previously described as “gondolas” within nanotubes, which are thought to be associated with the transport of large cargo [24] including organelles such as mitochondria.

### 3.2. Bridges Are Membrane-Bound and Contain Actin and Tubulin

**Figure 2:**
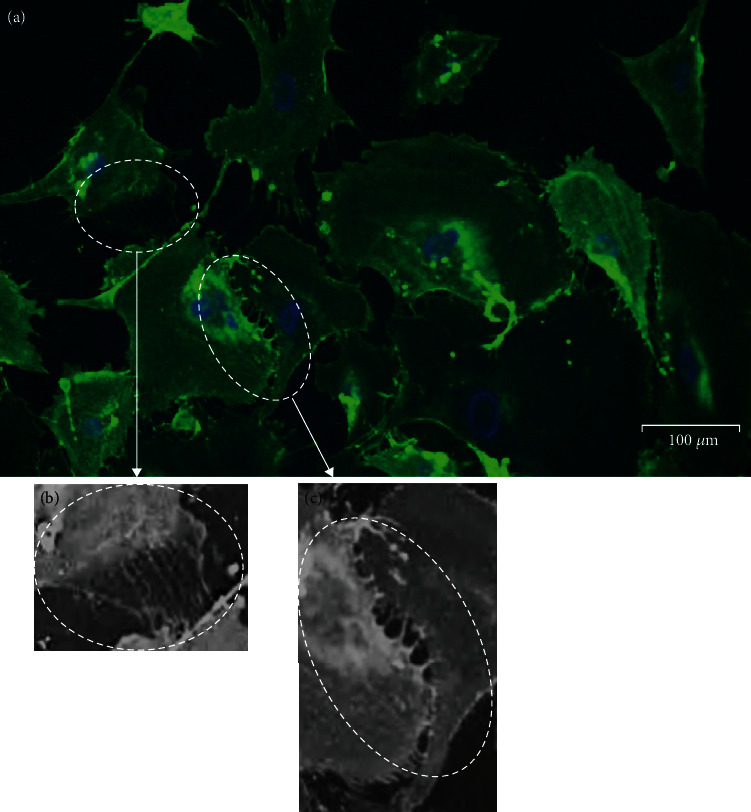
Intercellular bridges are membrane-bound and occur at a high frequency between senescent cells. (a) HF043 fibroblasts cultured to replicative senescence and imaged live with FITC-WGA (green) to highlight membrane-associated O-GlcNAc and NucBlue Live for DNA (blue). (b, c) Magnified images of thin bridges (b) and larger diameter bridges (c). Images in (b, c) have been recoloured in grayscale and sharpness-enhanced to show the bridges more clearly.

To determine whether the observed senescent cell bridges are membrane-bound, we stained cells with FITC-conjugated lectin wheat germ agglutinin (WGA) to assess the presence of the sugar O-GlcNAc, which is prevalent on mammalian membranes; DNA was counterstained with NucBlue Live. From Figure 2(a), multiple membrane-bound (WGA-positive) protrusions can be seen to extend between the senescent cells towards their neighbours, in such a way that the cells appear to form a “network” of interconnected cells rather than discrete cells. The diameter of such connections varied from ultrafine bridges (Figure 2(b)) reminiscent of “thin” TNTs to larger diameter bridges (Figure 2(c)) that appear consistent with previously described “thick” TNTs [7].

**Figure 3:**
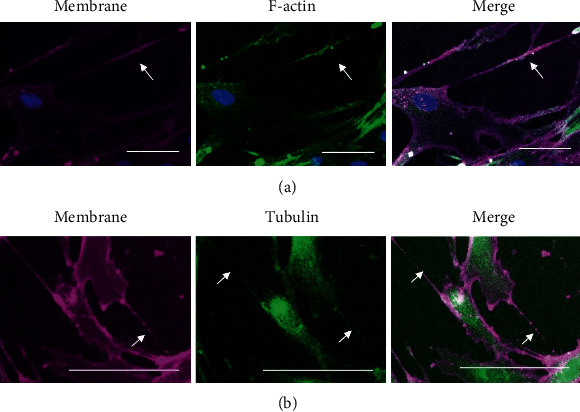
Intercellular contacts between senescent cells contain actin and tubulin. Replicatively senescent HF043 fibroblasts (CPD > 90) were (a) fixed and stained with rhodamine-WGA (membrane, purple), FITC-phalloidin (F-actin, green), and NucBlue Live (DNA, blue) or (b) imaged live with rhodamine-WGA (membrane, purple) and Tubulin Tracker Green (microtubules, green) prior to analysis by fluorescence microscopy. *n* = 3, representative images shown. Arrows indicate examples of intercellular bridges. Images have been false-coloured from the original red/green in Fiji to improve dye discrimination. Scale bar 100 *μ*m.

To determine whether these structures represent actin-stabilised tunnelling nanotubes (TNTs), formaldehyde-fixed cells were costained with rhodamine-WGA (for membranes) and FITC-phalloidin for actin and counterstained with NucBlue Live for DNA (Figure 3(a)) or imaged live with Tubulin Tracker Green to identify polymerised tubulin (Figure 3(b)). Fluorescence microscopy demonstrated that the intercellular bridges contain actin (Figure 3(a)) and tubulin (Figure 3(b)); in both cases, the bridges stained positively with rhodamine-WGA, demonstrating the presence of O-GlcNAc, orthogonally confirming with a different dye our observations in Figure 2. These data suggest that senescent fibroblasts form intercellular nanotubes that contain actin and/or tubulin.

### 3.3. Mitochondria Are Present in Senescent Cell TNTs

As signalling hubs and the major source of oxidative stress, mitochondria play an important role in senescence [25]. Senescent cells exhibit dramatic increases in mitochondrial load [21], possibly to compensate for mitochondrial dysfunction, and the mitochondrial network becomes increasingly reticular through resistance to mitochondrial fission and mitophagy [26].

**Figure 4:**
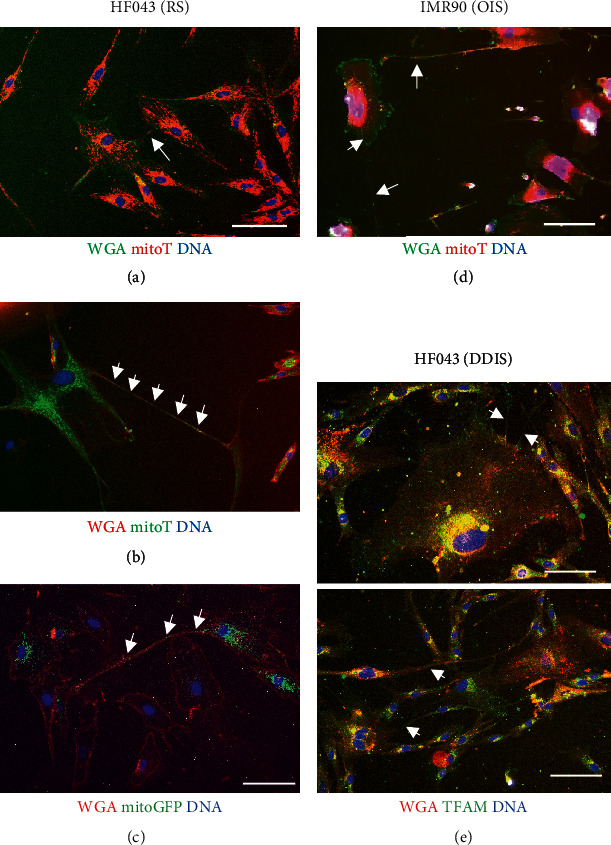
Mitochondria are present within senescent intercellular contacts. (a–c) Replicatively senescent HF043 fibroblasts were stained with (a) fluorescein-WGA and MitoTracker Red (mitoT), (b) rhodamine-WGA and MitoTracker Green (mitoT), and (c) rhodamine-WGA and CellLight Mitochondria-GFP BacMam (mitoGFP). (d) IMR90 ER:RAS cells induced to undergo oncogene-induced senescence (OIS) by 7 d treatment with 4-OHT were stained with fluorescein-WGA and MitoTracker Red (mitoT). Cells in (a–d) were imaged live. (e) HF043 fibroblasts treated for 7 d with mitomycin C to drive DNA damage-induced senescence (DDIS) were fixed and stained with anti-TFAM primary antibody, with Alexa Fluor 488 secondary antibody and rhodamine-WGA. Arrows indicate mitochondria within contacts. *n* ≥ 3. DNA stained with NucBlue Live. Scale bar 100 *μ*m.

To ask whether the senescent cell TNTs are capable of transporting mitochondria, we labelled mitochondria in senescent fibroblasts (RS and DDIS in HF043 and OIS in IMR90, as above) with fluorescent dye. To avoid labelling bias, we compared a variety of mitochondrial dyes including the live stains MitoTracker Green, MitoTracker Red, and CellLight Mitochondria-GFP BacMam, where GFP is localised to mitochondria through fusion of GFP with the leader sequence from E1 alpha pyruvate dehydrogenase. We also examined staining patterns of the mitochondrial transcription factor TFAM in fixed samples. In all cases, cell membranes were stained with fluorescent WGA (dye colour indicated in labels of Figure 4).

As shown in Figure 4, we observed small foci (Figures 4(a) and 4(c)) or even reticular networks (Figures 4(b) and 4(d)) positive for mitochondrial staining in a substantial proportion of intercellular bridges, irrespective of the mitochondrial stain used, the mode of senescence induction, or whether cells were imaged live or fixed, showing the presence of mitochondria within TNTs of senescent cells.

### 3.4. Senescent Cell Tunnelling Nanotubes Support Intercellular Mitochondrial Transport

The mitochondria present in intercellular bridges may arise simply through a chance peripheral distribution at the time and subcellular location of bridge formation; alternatively, they could represent cargo being transported through the bridges, as previously suggested [12]. Hence, to assess whether the observed mitochondria were static or mobile within these intercellular bridges, time-lapse fluorescence microscopy of replicatively senescent HF043 fibroblasts stained with MitoTracker Red was conducted.

**Figure 5:**
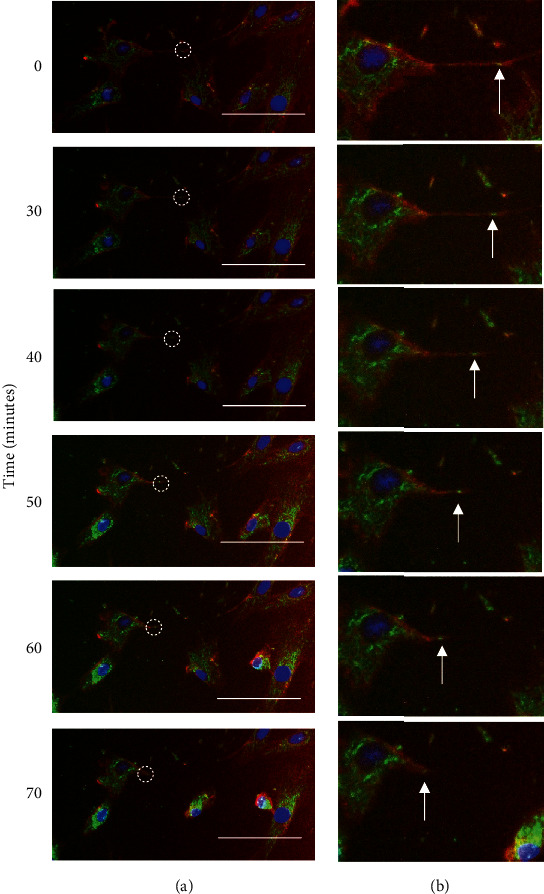
Mitochondria are motile within senescent intercellular bridges. (a) Replicatively senescent HF043 fibroblasts were stained with MitoTracker Green, rhodamine-WGA (red), and NucBlue Live (for DNA) and imaged live using time-lapse fluorescence microscopy, with time points following the start of the observation period indicated in minutes. The white dotted circle highlights a MitoTracker Green-positive punctum within a TNT. Representative images shown of *n* = 3 experiments. Scale bar 100 *μ*m. (b) Magnification of TNT with position of moving MitoTracker-positive puncta indicated by arrows (see also Supplementary Video (available here)).

Mitochondrial motility was observed within minutes of staining, and mitochondria were seen to track along intercellular bridges, as seen in representative still images of time-lapse microscopy (Figure 5; see also Supplementary Video (available here)). All movement detected for individual mitochondrial puncta was unidirectional, with an average speed of ~1 *μ*m/minute within the observation period; the total distance travelled by the mitochondria in the example shown in Figure 5 was ~60 *μ*m within 70 minutes.

These observations suggest that mitochondria are able to move within senescent intercellular bridges over the timescale of minutes to hours. The observation of unidirectional movement suggests that mitochondrial motility may be regulated, rather than occurring by passive diffusion, possibly involving motor proteins acting on the underlying cytoskeletal framework of actin and/or tubulin.

### 3.5. Co-culture Demonstrates Transfer of Mitochondria between Cells

**Figure 6:**
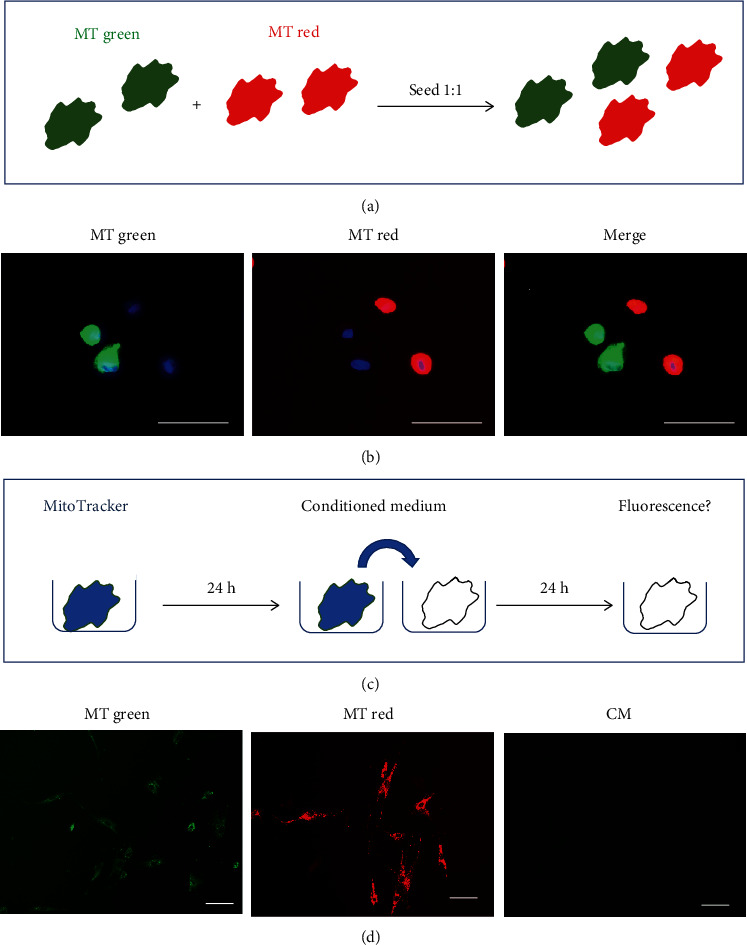
Co-culture assay for analysis of intercellular mitochondrial transfer. (a) Schematic of the co-culture setup. HF043 fibroblasts were stained with MitoTracker Green (MT green) or MitoTracker Red (MT red), washed thoroughly, and then seeded into co-culture at a 1 : 1 ratio. (b) Cells were imaged immediately after co-plating (proliferating cells shown). (c) Schematic of assay for dye leakage: cells were stained for 30 min with MitoTracker Green or Red, media replaced, and incubated for 24 h to generate conditioned medium (CM). This CM was then harvested and incubated with unstained cells for 24 h prior to fluorescence microscopy. (d) Representative images of proliferating cells stained with MitoTracker Green or MitoTracker Red or unstained cells incubated for 24 hours with conditioned media (CM) harvested from stained cells. *n* ≥ 3. Scale bar 100 *μ*m.

To investigate whether this putative mitochondrial transport resulted in the transfer of mitochondria between cells, we next conducted a co-culture assay where separate populations of cells were stained with MitoTracker Green and MitoTracker Red, respectively, then thoroughly washed before harvesting and co-seeding at a 1 : 1 ratio (schematic shown in Figure 6(a), with images of co-seeded cells immediately after plating shown in Figure 6(b)).

To exclude the possibility of MitoTracker dye carry-over between different cell populations in co-culture experiments through dye leakage into the medium, conditioned medium (CM) controls were included in each experiment whereby media were harvested after 24 hours of exposure to stained cells (with either MitoTracker Green or MitoTracker Red, shown simply as “MitoTracker” in schematic Figure 6(c)), followed by incubation with unstained cells for 24 hours with inspection for any fluorescent signal by microscopy; no evidence of dye leakage was detected (Figure 6(d)).

**Figure 7:**
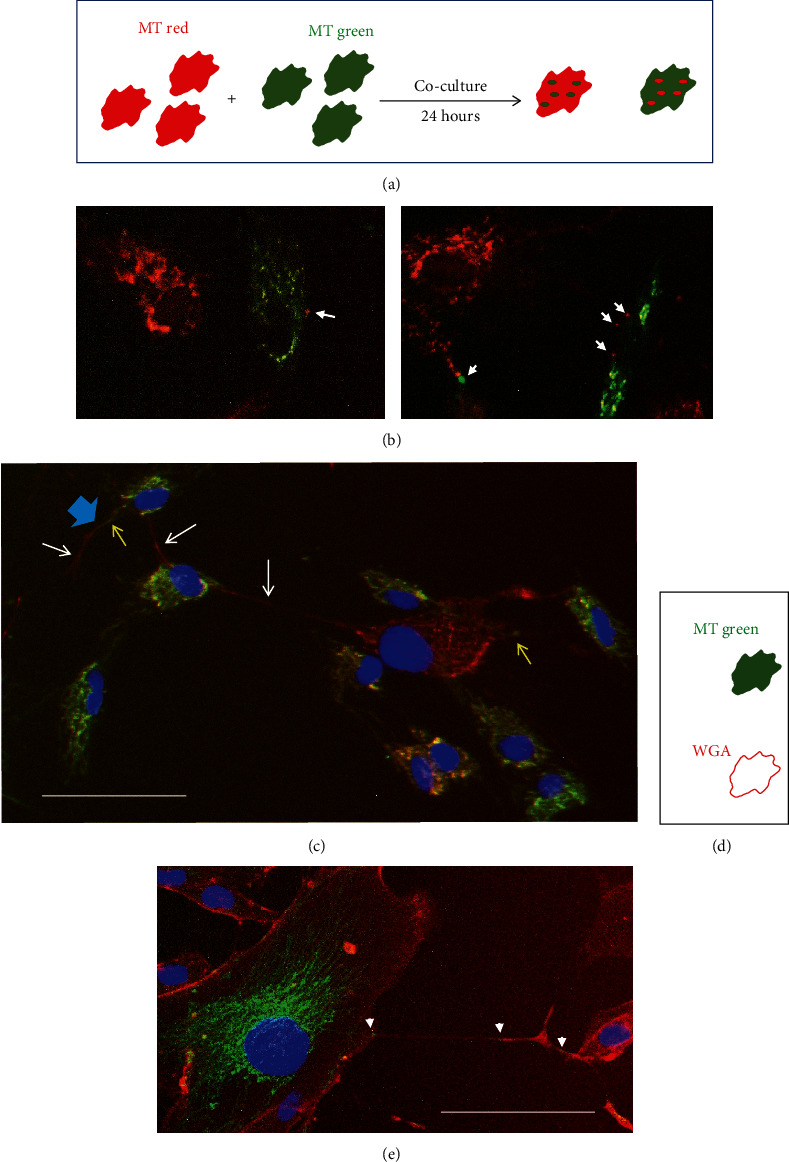
Mitochondrial transfer between co-cultured cells. (a) Schematic of co-culture experiment. One cell population is labelled by incubation with MitoTracker (MT) red and another with MT green for 30 min. Following washing and harvesting, the differently labelled cells are seeded at a 1 : 1 ratio and co-cultured for 24 h prior to fluorescence microscopy analysis. (b) Examples of mitochondrial puncta arising through the transfer between co-cultured cells (white arrows). (c) Example of TNTs that appear to contain reticular mitochondria. White arrows indicate TNTs arising from cells labelled with MitoTracker Red, and yellow arrows indicate those arising from cells labelled with MitoTracker Green. The blue arrow indicates a TNT that appears to be formed by the fusion of a bridge between two differently labelled cells. (d) Schematic showing co-culture between cells labelled with MitoTracker (MT) Green and unlabelled cells. (e) Representative image of co-culture with an MT green-labelled cell that has undergone mitomycin C-induced senescence (DDIS) and unlabelled proliferating cells (rhodamine-WGA was used subsequently to highlight cell membranes). White arrows indicate the presence of green mitochondrial puncta being transferred to a cell without mitochondrial labelling. Scale bar 100 *μ*m.

Having optimised and verified labelling and co-culture conditions as shown in Figure 6, we then assessed whether mitochondria were transferred between differentially labelled cell populations. One population of HF043 cells was labelled with MitoTracker Red (MT red) and another with MitoTracker Green (MT green), harvested, co-seeded at 1 : 1, and then imaged after 24 hours. Any transfer of mitochondria between cells of different populations might be visualised as discrete puncta of the opposite dye colour, as shown in the schematic (Figure 7(a)). Indeed, such puncta were detected in a number of co-cultured cells (white arrows, Figure 7(b)), irrespective of the MitoTracker dye used. In co-cultures of senescent cells, we also observed what appear to be mitochondrial reticular networks extending through TNTs between cells over considerable distances (>100 *μ*m, white arrows Figure 7(c)), with large senescent “donor” cells also appearing to accept mitochondria from oppositely stained cell populations (yellow arrows, Figure 7(c)). We further observed instances where TNTs were stained with both red and green, suggesting formation by fusion of TNTs extruded from separate cells (blue arrow, Figure 7(c)). Mitochondria were also transferred through TNTs from labelled DDIS cells to unlabelled proliferating cells (Figures 7(d) and 7(e)).

### 3.6. Mitochondria Pass from Senescent Cells to Proliferating Cells and Vice Versa

**Figure 8:**
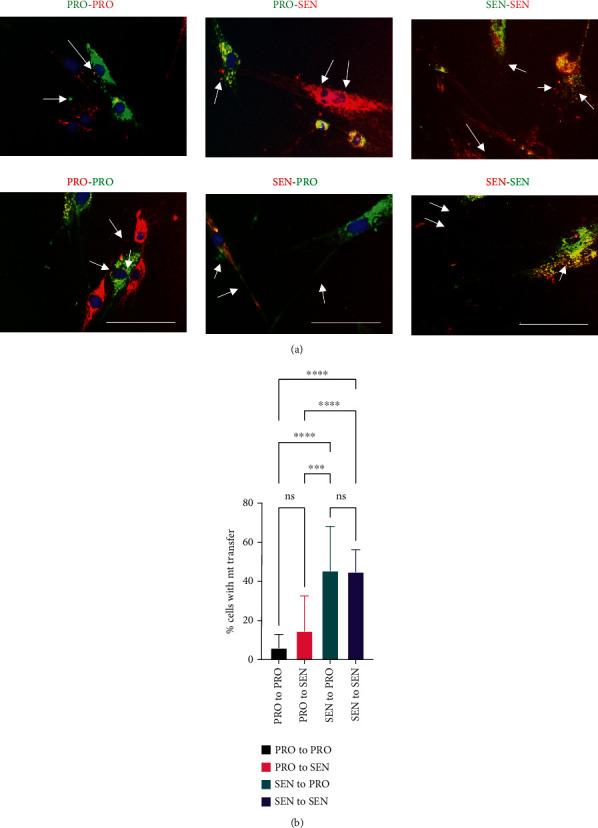
Intercellular mitochondrial transfer occurs between both proliferating and senescent cells. (a) Co-cultures of proliferating (PRO) or replicatively senescent (SEN) HF043 fibroblasts pre-stained with MitoTracker Green or Red were set up as in Figure 6(a) and imaged following 24 hours of co-incubation. Arrows indicate intercellular bridges or puncta of transferred mitochondria. DNA was stained with NucBlue Live immediately prior to analysis. Scale bar 100 *μ*m. (b) Quantification of the mitochondrial transfer between proliferating (PRO) and senescent (SEN) cells. One-way ANOVA, ns = not significant. ^∗∗∗^*p* = 0.0002, ^∗∗∗∗^*p* < 0.0001 (*n* = 16 for PRO-PRO and SEN-SEN, *n* = 9 for each of PRO-SEN and SEN-PRO).

Intercellular mitochondrial transfer has been hypothesised as a rescue mechanism for stressed cells, including cancer cells that have lost mitochondrial functionality [12]. To determine whether, like stressed cancer cells, senescent cells can accept mitochondria from healthy proliferating cells, we conducted co-culture experiments between populations of proliferating and senescent cells, with “red”-stained proliferating cells co-cultured with “green”-stained proliferating cells (PRO-PRO), “red” senescent cells with “green” senescent cells (SEN-SEN), or proliferating and senescent (PRO-SEN) co-cultures (using both colour combinations to eliminate any effect of dye bias)—dye colours are indicated in labels of Figure 8. Nuclei were counterstained using the live dye NucBlue Live prior to imaging.

After 24 hours of co-culture, we observed puncta of oppositely stained mitochondria within both proliferating and senescent fibroblasts, regardless of which population was stained with MitoTracker Green or MitoTracker Red (Figure 8(a)), as well as mitochondria-loaded bridges spanning alternately stained “red” and “green” cells, suggesting that mitochondria have been transferred between different cells, with proliferating and senescent cell populations able to act both as mitochondrial donors and acceptors. However, quantification of the extent of mitochondrial transfer shows that proliferating cells rarely act as mitochondrial donors, both in co-cultures with other proliferating cells and when co-cultured with senescent cells (Figure 8(b)). However, mitochondrial transfer by senescent cells occurred at equivalent levels whether the acceptor cells were proliferating or senescent. We verified that mitochondrial transfer direction was not an artefact of the MitoTracker dye used (Supplementary Figure S4), supporting the conclusion that senescent cells are major mitochondrial donors.

Taken together, our results suggest that tubulin- and actin-based intercellular bridges may constitute an important mechanism of mitochondrial transfer and thus intercellular communication for senescent cells, and this transfer may occur from senescent to proliferating cells within a tissue.

### 3.7. Direct Intercellular Communication in Senescence Is Regulated by mTOR and CDC42 Signalling

Intercellular bridge structures between cancer cells have been reported to be regulated by mTOR signalling [6], while actin regulatory factors have been implicated in senescent cell TNTs [16]. We therefore tested whether the intercellular bridges observed here are regulated by CDC42 or mTOR signalling, using the pharmacological inhibitors CASIN and AZD8055 to inhibit CDC42 and mTOR, respectively, in both the senescent and proliferating cell populations. As an ATP-competitive mTOR inhibitor, AZD8055 impacts not only pathways regulated by mTORC1 (such as translation and autophagy) but also those controlled by mTORC2, including actin polymerisation (e.g., [21]).

**Figure 9:**
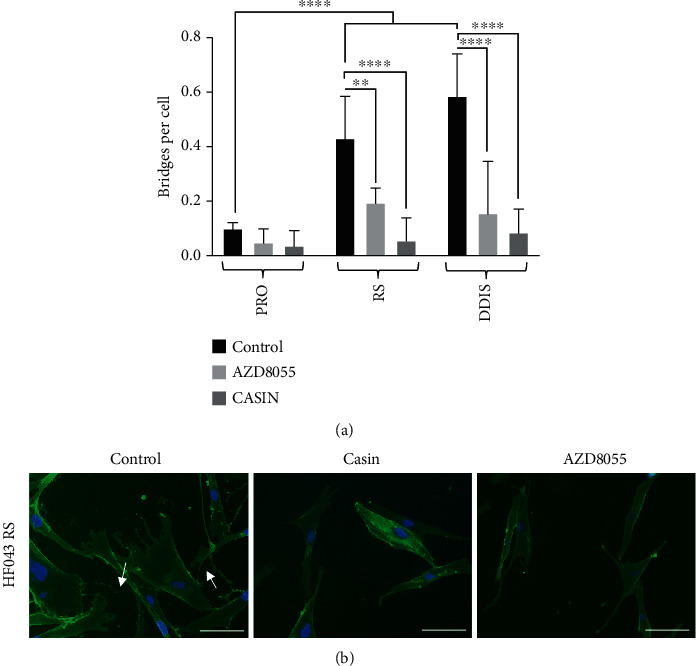
CDC42 and mTOR signalling are required for the formation of intercellular bridges in senescent cells. (a) HF043 fibroblasts that had undergone replicative senescence (RS, cumulative population doubling ≥ 90), DNA damage-induced senescence (DDIS, following 7-day treatment with 20 *μ*M etoposide), and proliferating (PRO) control cell populations at low cumulative population doubling (CPD) were treated with the mTOR inhibitor AZD8055 (70 nM) or the CDC42 inhibitor CASIN (2 *μ*M) for 24 hours before fixation and staining with fluorescein-WGA (green, for membranes) and NucBlue Live (for DNA). Intercellular bridges were manually quantified from >50 cells per replicate (*n* = 3). ^∗∗^*p* < 0.05, ^∗∗∗^*p* < 0.001. (b) Representative images of the control and drug-treated replicatively senescent fibroblasts. Arrows indicate intercellular bridges (TNTs). Scale bar = 100 *μ*m.

Populations of proliferating or senescent HF043 fibroblasts which had undergone either replicative senescence (RS) or DNA damage-induced senescence (DDIS) through 7-day treatment with 20 *μ*M etoposide were exposed to the CDC42 inhibitor CASIN at 2 *μ*M, pan-mTOR inhibitor AZD8055 [21] at 70 nM, or DMSO (vehicle control) for 24 hours prior to fixation with formaldehyde. Cells were then stained with fluorescein-WGA for cell membranes and NucBlue Live for nuclear DNA. The number of intercellular bridges connected at both ends was counted manually, and the number per cell was determined by normalising against nuclear number (Figure 9(a)).

We observed significantly more intercellular bridges within senescent (RS and DDIS) compared with proliferating control populations (PRO, Figure 9(a)), with an average of ~0.5 bridges per replicatively senescent (RS) cell. While a substantial proportion of cells exhibited several connections, others had none. Notably, disruption of CDC42 signalling upon CASIN treatment, or inhibition of mTOR signalling with AZD8055, each significantly reduced the number of bridges between senescent cells, both in replicatively senescent populations and those induced by DNA damage (Figure 9(a)); representative fluorescence images of treated RS cells are shown in Figure 9(b). The very low frequency of bridges on treatment precluded analysis of the number of mitochondrial puncta transferred. The observed reduction in bridges was not due to toxicity at the drug doses used, as cell numbers were not diminished (as assessed by manual inspection). However, drug treatment did lead to lower apparent biochemical reducing capacity as measured by alamarBlue assays (Supplementary Figure S5), potentially because of decreased cell size following drug-induced structural rearrangements of the actin cytoskeleton (e.g., [21]). We further observed that the reduction in bridge number occurred in a dose-dependent manner (Supplementary Figure S6), strongly suggesting that mTOR and CDC42 are centrally important in TNT formation and/or stability.

## 4. Discussion

Entry into senescence is accompanied by development of a number of distinctive phenotypes including epigenetic alterations, lysosomal and mitochondrial dysfunction, proinflammatory secretion through the SASP, and morphological alterations including hypertrophy and changes to actin organisation networks. Furthermore, senescent cells are highly communicative, alerting immune cells to their presence for clearance [27] and regulating the proliferative capacity of surrounding healthy or cancerous cells positively and negatively [28, 29]. While this interplay has largely been attributed to secretion of exosomes and SASP factors, we show a high prevalence of intercellular bridges between senescent cells that may also be important in mediating these effects.

### 4.1. Supercellularity in Senescence?

While intercellular nanotubes appear to constitute a highly localised form of communication, it is important to note that these bridges often do not just connect two cells but instead can form networks extending direct cell communication over larger distances. Indeed, we have observed cellular networks that appear almost syncytial in nature, with multiple connections between multiple cells (e.g., Figure 2). Hence, by allowing the transport of cargo including whole organelles such as mitochondria between connected cells, intercellular nanotubes may create temporary syncytia or “supercellularity.” Notably, syncytia are permanently created through cell-cell fusion, an essential event in a number of developmental and physiological processes, such as mammalian muscle and osteoclast function, and in the formation of the placental syncytiotrophoblast, a giant cell of ~12m^2^ surface area [30]. Intriguingly, cell fusion events have been linked to cellular senescence: the placental syncytiotrophoblast itself shows a number of markers of senescence [30], while expression of fusogens such as endogenous retroviral ERVWE1 or measles virus induces cell fusion and induction of cell cycle arrest and senescence [31]. Through induction of senescence, fused cells co-ordinate a protection strategy to prevent division of multinucleated cells, while maintaining cell viability through resisting apoptosis, which may be important within the placental syncytiotrophoblast throughout pregnancy [31] and in other physiological contexts such as bone and muscle. It is interesting to speculate that through direct intercellular nanotubes, senescent cells could also relay danger signals to neighbouring cells and co-ordinate induction of paracrine senescence, just as cell fusion may induce senescence in syncytia. It will be of interest to assess the degree of electrical continuity between such connected senescent cells, as direct cytoplasmic bridges large enough for passage of mitochondria negate the need for other junctional structures such as gap junctions. Membrane potentials have been reported to be very low in senescence [32], and this may have implications for the low propensity of senescent cells to undergo apoptosis. Direct cell-cell contacts leading to supercellularity are therefore of particular interest in senescence and in development of senomodifying therapies.

### 4.2. Pathological Relevance of Direct Intercellular Contacts

While the data we present here suggest that a substantial proportion of senescent cells formed direct cell-cell bridges which can mediate intercellular mitochondrial transfer, it is important to note that these data were acquired in 2-dimensional *in vitro* cell culture of primary human skin and lung fibroblasts. While further work is undoubtedly required to probe the physiological relevance of these findings, previous *in vivo* work has shown that intercellular contacts can both rescue compromised cells and spread disease or infection, suggesting context-dependent function or exploitation by invading pathogens. Indeed, TNTs between tumour cells can play important roles in pathogenesis and invasion, for example, through intercellular transfer of the P-glycoprotein drug efflux pump to propagate multidrug resistance [33], together with intercellular mitochondrial transport for cellular rescue. TNTs have been implicated in the intercellular spread of pathogens such as HIV [34], influenza [35], and herpes viral particles as well as prions [36]. Importantly, in the context of age-related diseases, protein aggregates implicated in neurodegenerative diseases, including polyglutamine aggregates [37], *α*-synuclein [11, 38], and tau [39], have also been reported to be passed between cells through TNTs. The impact of intercellular tunnelling nanotubes is therefore likely to be determined by both the transferred cargo as well as the types and states of the connected cells.

### 4.3. Possible Roles of Mitochondrial Transfer through TNTs

Mitochondria are important signalling hubs for oxidative stress, apoptosis, and senescence. While we note here transfer of mitochondria between cells, we did not address the functional status of the transferred mitochondria. Senescent cells are known to accumulate dysfunctional mitochondria, and previous reports suggest that healthy mitochondria may be preferentially transferred to stressed cells [12]. Measuring the O_2_ consumption and activity of individual ETC components in senescent and proliferating cells following co-culture would therefore be informative as to whether healthy or dysfunctional mitochondria are preferentially transferred. Another important line of enquiry is whether the nanotube structures that senescent cells make with NK cells [12] also participate in intercellular mitochondrial transfer, as well as analysing the impact of direct interaction between senescent and proliferating cells, e.g., through analysis of proliferation capacity in co-cultures with direct contact compared with barrier trans-well formats. Moreover, it will be important to investigate the role of these intercellular connections in a physiologically relevant model, for example, in 3D culture formats such as hydrogels of appropriate rigidity reflecting “young” and “old” tissue structures. Indeed, recently published data analysing TNT formation in mesenchymal stem cell (MSC) spheroids showed intercellular transfer of cytosolic dyes, and intriguingly, inclusion of low passage MSCs with high passage cells was seen to abrogate p16 expression in spheroids, dependent on TNT formation. Hence, in this context, TNTs appear to rescue the proliferation potential of high passage cells, potentially overcoming senescence induction [16].

### 4.4. Role of Cell-Cell Communication in Senescence

While senescent cells are known to accumulate in tissues with chronological age and within tumours, they also play important roles in development, tissue regeneration, and wound healing. Furthermore, the profile of cytokines, chemokines, matrix remodelling enzymes and growth factors secreted in the SASP is highly heterogeneous between cell types and modes of senescence induction, perhaps underpinning the range of different paracrine responses to local induction of senescence. In these different physiological contexts, it is highly likely that senescent cells could exert a number of different impacts through direct intercellular bridges. Indeed, it is possible that within a tumour, intercellular bridges could participate in both rescuing cancer cells with dysfunctional mitochondria, but also in facilitating communication between the senescent and immune cells to promote immune surveillance [16]. It is also important to note that senescent cells have actually been shown to evade immune surveillance, for example, by HLA-E upregulation [40]; thus, TNTs may enable evasion of immune detection through shuttling of surface markers. Moreover, direct connections between senescent cells and proliferating neighbours may provide an additional means for spread of senescence—possibly even in the absence of secreted SASP factors that can induce bystander senescence. Therapies that focus on suppressing the SASP may therefore not be sufficient to ameliorate damaging aspects of senescent cell accumulation in cancer and ageing, and may additionally need to include approaches to disrupt direct cell-cell contacts through TNTs.

### 4.5. Targeting Intercellular Tunnelling Nanotubes in Human Disease

Proteins that promote actin polymerisation and stabilisation play an important role in facilitating direct cell-cell contact via membrane-bound bridges [16]. Using co-culture assays and live cell imaging, we report here that not only do these bridges increase in frequency in senescence resulting from various stresses (replicative exhaustion, DNA damage, or oncogene activation), but that they are regulated by mTOR and CDC42 signalling, and facilitate direct intercellular mitochondrial transfer. Importantly, CDC42 may act downstream of constitutive mTOR activity in senescence.

While intercellular bridges may play important roles in physiological processes requiring cell-cell communication, such as aiding NK-mediated recognition of senescent cells [31], the pathological effects of cell-cell bridges may be amenable to therapeutic intervention. CDC42 signalling, implicated in the actin organisation network, is reported to be elevated in senescent versus proliferating cells and to promote premature ageing [41] and, as we show here, is necessary for TNT formation and/or stability. Actin cytoskeletal rearrangements, regulated by mTOR- and CDC42-dependent pathways, are also likely to play a role in the enlarged, flattened morphology observed for senescent cells *in vitro* [21], as well as enlargement *in vivo* [42], and our results show that senescent cells have higher numbers of TNTs than proliferating cells.

The importance of CDC42 and mTOR in senescence may therefore extend beyond regulating the SASP [43, 44] and the biogenesis and secretion of exosomes [45], to promoting direct intercellular communication through the formation of direct cell-cell communication channels. Consequently, our findings highlight mTOR signalling as critical in directing the non-cell-autonomous roles of cellular senescence and further emphasise its validity as a therapeutic target for senomodifying therapies, building on reports of beneficial effects of mTOR inhibitors on senescence phenotypes *in vitro* [21] and longevity and health *in vivo* [46, 47]. It is tempting to speculate that the utility of mTOR inhibitors in a number of age-related diseases (reviewed in [48]) including Alzheimer's disease ([49]; reviewed in [50]) and immune senescence [51], as well as their ability to improve ageing human skin structure [52], may be at least in part through actin cytoskeletal modulation that impacts TNT formation.

Reducing TNT formation or stability through mTOR or CDC42 inhibition may be a useful therapeutic approach in cancer as well as in ageing, for example, through blocking the transfer of healthy mitochondria from bystander cells to rescue dysfunctional tumour cells, or through blocking the spread of drug resistance channels. Indeed, neutralising antibodies against the adhesion molecule ICAM-1 in T cell acute lymphoblastic leukaemia were shown to block the transfer of healthy mitochondria from MSCs, thereby causing an increase in chemotherapy-induced cancer cell death [53]. This approach may also be important in preventing the spread of pathogens such as HIV or protein aggregates implicated in neurodegeneration: inhibiting nanotube formation with latrunculin B was shown to prevent the spread of *α*-synuclein through healthy astrocytes in a model of Parkinson's disease propagation [54].

Alternatively, it may be possible to exploit the beneficial properties of nanotubes, for example, in treating diseases of organelle dysfunction or by using intercellular mitochondrial transfer as a rescue strategy for compromised cells during stroke, when ischaemic stress conditions may stimulate nanotube formation. In such circumstances, it may be possible to further promote nanotube formation by supplying a treatment that induces ROS, stabilizes microtubules or actin networks, or through inducing increased expression of trafficking adaptors in the mitochondria “donor” cells. It may even be possible to exploit intercellular nanotubes as a drug delivery pathway in cancer therapy, as nanoparticles have been shown to be loaded into and move through TNTs [55].

## 5. Conclusions

In conclusion, we have demonstrated here a high prevalence of intercellular bridges (also called tunnelling nanotubes (TNTs)) between senescent cells, which are membrane-bound and supported by a cytoskeleton containing actin and possibly also microtubules. Such bridges allow transfer of large cargo including mitochondria between cells. The dependence of such bridges on mTOR and CDC42 makes them amenable to small molecule intervention, providing additional therapeutic strategies to modulate senescent cell behaviour in both age-related diseases and cancer.

## Data Availability

Data are available from the corresponding author upon reasonable email request.

## Supplementary Materials

Supplementary Materials Supplementary Video: time-lapse video microscopy of mitochondrial transfer via TNTs in replicatively senescent HF043 fibroblasts stained with MitoTracker Green, rhodamine-WGA, and NucBlue Live (for DNA). Scale bar 32 *μ*m. Note: still images from this video are shown in Figure 5. Supplementary Figure S1: growth curve of primary skin fibroblasts. HF043 cells were continuously cultured until replicative senescence at cumulative population doubling (CPD) ≥ 86. Each point represents harvesting and reseeding of cells under continuous cultivation, when cell numbers are counted to calculate CPD. Supplementary Figure S2: markers of senescence. Proliferating and senescent cells were verified by both morphological analysis under phase contrast microscopy and staining for senescence-associated beta-galactosidase (SA-*β-*gal). For replicative senescence, proliferating HF043 cells at CPD < 40 and senescent cells at CPD ~ 87. For DNA damage-induced senescence, proliferating HF043 skin fibroblasts at low CPD were treated with etoposide (see Materials and Methods); for oncogene-induced senescence, proliferating IMR90 ER:RAS cells were treated with 4-hydroxytamoxifen (4-OHT) to induce *RAS* expression. CTRL = control. Scale bar 50 *μ*m. Supplementary Figure S3: markers of senescence. (A, B) IL-6 SASP factor secretion is elevated in senescent versus proliferating cells. (A) Standard curve for IL-6 ELISA using purified recombinant IL-6. (B) Measurement of IL-6 in proliferating (PRO) and replicatively senescent (SEN) cells by ELISA (*n* = 3, mean ± SD). (C) Upregulation of p21 in senescent cells. Representative western blotting of p21^CDKN1^ and loading control GAPDH in proliferating (PRO) cells with (+) or without (-) DNA damaging agent etoposide to induce DDIS, and replicatively senescent (SEN) cells without etoposide treatment. Supplementary Figure S4: senescent cells are the major donors for mitochondrial transfer. Cells were prelabelled with MitoTracker Green or MitoTracker Red as in the main text and co-cultured at a 1 : 1 ratio. (A) Enlarged areas from cells in Figures 4 and 7, showing transfer of both the red and green mitochondria between cells. Scale bar 50 *μ*m. (B) Transfer of mitochondria quantified by MitoTracker dye colour of the donor and recipient; bars coloured by the MitoTracker label of recipient cells; the MitoTracker label of the donor and recipient is indicated by colour below each column (MitoTracker Green shown in green and MitoTracker Red shown in red). P = proliferating; S = replicatively senescent. One-way ANOVA. ns = not significant. ^∗∗∗^*p* = 0.0002. (C) Violin plot of quantification of mitochondrial donation, according to the percentage of recipient cells stained with MitoTracker Red (R) showing donated MitoTracker Green (G) foci (%R cells with G mt) and the percentage of recipient cells stained with MitoTracker Green (G) showing donated MitoTracker Red (R) foci (i.e., %G cells with R mt). Two-way ANOVA. ns = not significant. ^∗∗∗∗^*p* < 0.0001. Mean, 25^th^ and 75^th^ centiles, and individual data points are shown. mt = mitochondria. Supplementary Figure S5: dose-response curves for cells treated with (A) AZD8055 or (B) CASIN at concentrations from 0 to 10,000 nM. Cell reducing capacity, often used as a proxy for cell viability, was assessed by alamarBlue staining. Note: 0 nM not shown as data are plotted on a log scale; values are normalised to zero drug control, taken as 100%. Drug doses were tested on proliferating (PRO) cells as well as replicatively senescent (RS) and DDIS cells induced by etoposide treatment (ETOP). Mean and SD are shown from *n* = 3. Supplementary Figure S6: intercellular bridge formation is sensitive to CDC42 inhibition in a dose-dependent manner. HF043 fibroblasts at CPD 71 were incubated with 0, 1, 2, and 5 *μ*m CASIN, an inhibitor of CDC42 for 24 h, and then stained with FITC-WGA (green), rhodamine-phalloidin (actin, red), and NucBlue Live for DNA (blue). Scale bar 100 *μ*m.
