# Supplementary material for: Intercellular Transfer of Mitochondria between Senescent Cells through Cytoskeleton-Supported Intercellular Bridges Requires mTOR and CDC42 Signalling
Source: Oxid Med Cell Longev. 2021 Jul 31;2021:6697861. doi: 10.1155/2021/6697861 (PMC8349290; doi:10.1155/2021/6697861)
Supplement: Supplementary Materials — Supplementary Video: time-lapse video microscopy of mitochondrial transfer via TNTs in replicatively senescent HF043 fibroblasts stained with MitoTracker Green, rhodamine-WGA, and NucBlue Live (for DNA). Scale bar 32 μm. Note: still images from this video are shown in Figure 5. Supplementary Figure S1: growth curve of primary skin fibroblasts. HF043 cells were continuously cultured until replicative senescence at cumulative population doubling (CPD) ≥ 86. Each point represents harvesting and reseeding of cells under continuous cultivation, when cell numbers are counted to calculate CPD. Supplementary Figure S2: markers of senescence. Proliferating and senescent cells were verified by both morphological analysis under phase contrast microscopy and staining for senescence-associated beta-galactosidase (SA-β-gal). For replicative senescence, proliferating HF043 cells at CPD < 40 and senescent cells at CPD ~ 87. For DNA damage-induced senescence, proliferating HF043 skin fibroblasts at low CPD were treated with etoposide (see Materials and Methods); for oncogene-induced senescence, proliferating IMR90 ER:RAS cells were treated with 4-hydroxytamoxifen (4-OHT) to induce RAS expression. CTRL = control. Scale bar 50 μm. Supplementary Figure S3: markers of senescence. (A, B) IL-6 SASP factor secretion is elevated in senescent versus proliferating cells. (A) Standard curve for IL-6 ELISA using purified recombinant IL-6. (B) Measurement of IL-6 in proliferating (PRO) and replicatively senescent (SEN) cells by ELISA (n = 3, mean ± SD). (C) Upregulation of p21 in senescent cells. Representative western blotting of p21CDKN1 and loading control GAPDH in proliferating (PRO) cells with (+) or without (-) DNA damaging agent etoposide to induce DDIS, and replicatively senescent (SEN) cells without etoposide treatment. Supplementary Figure S4: senescent cells are the major donors for mitochondrial transfer. Cells were prelabelled with MitoTracker Green or MitoTracker Red as in the main [file 6697861.f1.zip › Supp FIg 2 FINAL.pdf]

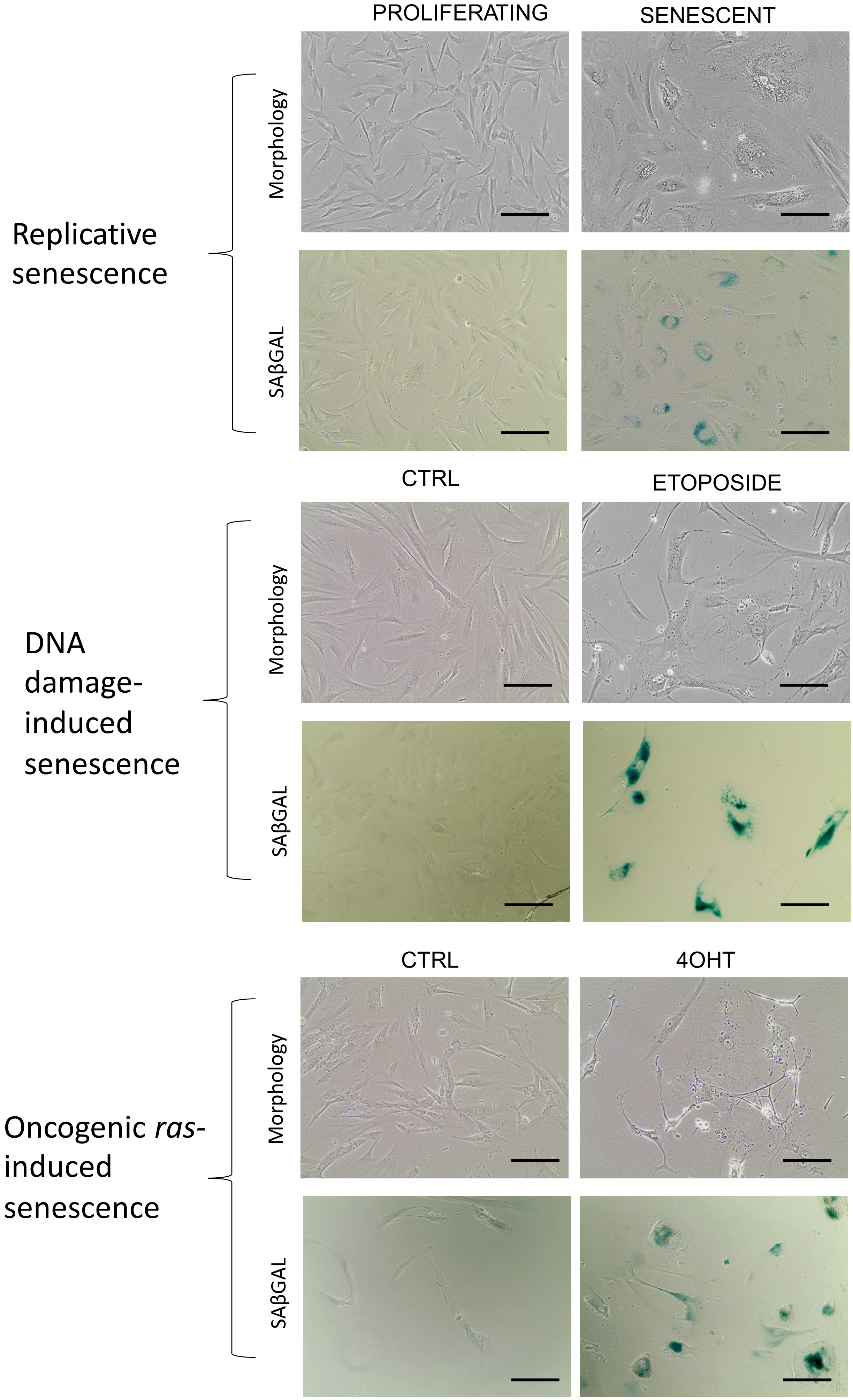
PROLIFERATING
SENESCENT
Replicative senescence
Morphology
SAβGAL
CTRL
ETOPOSIDE
DNA damage-induced senescence
Morphology
SAβGAL
CTRL
4OHT
Oncogenic *ras*-induced senescence
Morphology
SAβGAL
