# Supplementary material for: Intercellular Transfer of Mitochondria between Senescent Cells through Cytoskeleton-Supported Intercellular Bridges Requires mTOR and CDC42 Signalling
Source: Oxid Med Cell Longev. 2021 Jul 31;2021:6697861. doi: 10.1155/2021/6697861 (PMC8349290; doi:10.1155/2021/6697861)
Supplement: Supplementary Materials — Supplementary Video: time-lapse video microscopy of mitochondrial transfer via TNTs in replicatively senescent HF043 fibroblasts stained with MitoTracker Green, rhodamine-WGA, and NucBlue Live (for DNA). Scale bar 32 μm. Note: still images from this video are shown in Figure 5. Supplementary Figure S1: growth curve of primary skin fibroblasts. HF043 cells were continuously cultured until replicative senescence at cumulative population doubling (CPD) ≥ 86. Each point represents harvesting and reseeding of cells under continuous cultivation, when cell numbers are counted to calculate CPD. Supplementary Figure S2: markers of senescence. Proliferating and senescent cells were verified by both morphological analysis under phase contrast microscopy and staining for senescence-associated beta-galactosidase (SA-β-gal). For replicative senescence, proliferating HF043 cells at CPD < 40 and senescent cells at CPD ~ 87. For DNA damage-induced senescence, proliferating HF043 skin fibroblasts at low CPD were treated with etoposide (see Materials and Methods); for oncogene-induced senescence, proliferating IMR90 ER:RAS cells were treated with 4-hydroxytamoxifen (4-OHT) to induce RAS expression. CTRL = control. Scale bar 50 μm. Supplementary Figure S3: markers of senescence. (A, B) IL-6 SASP factor secretion is elevated in senescent versus proliferating cells. (A) Standard curve for IL-6 ELISA using purified recombinant IL-6. (B) Measurement of IL-6 in proliferating (PRO) and replicatively senescent (SEN) cells by ELISA (n = 3, mean ± SD). (C) Upregulation of p21 in senescent cells. Representative western blotting of p21CDKN1 and loading control GAPDH in proliferating (PRO) cells with (+) or without (-) DNA damaging agent etoposide to induce DDIS, and replicatively senescent (SEN) cells without etoposide treatment. Supplementary Figure S4: senescent cells are the major donors for mitochondrial transfer. Cells were prelabelled with MitoTracker Green or MitoTracker Red as in the main [file 6697861.f1.zip › Supplementary Figure legends.docx]

**Supplementary Video:** Time lapse video microscopy of mitochondrial transfer via TNTs in replicatively senescent HF043 fibroblasts stained with Mitotracker Green, rhodamine-WGA and NucBlue Live (for DNA) Scale bar 32 μm. Note still images from this video are shown in Figure 5.

**Supplementary Figure legends**

**Supplementary figure S1. Growth curve of primary skin fibroblasts.** HF043 cells were continuously cultured until replicative senescence at cumulative population doubling (CPD) ≥86. Each point represents harvest and reseeding of cells under continuous cultivation, when cell numbers are counted to calculate CPD.

**Supplementary Figure S2. Markers of senescence.** Proliferating and senescent cells were verified both by morphological analysis under phase contrast microscopy, and staining for senescence-associated beta galactosidase (SAβgal). For replicative senescence, proliferating HF043 cells at CPD<40, senescent cells at CPD~87. For DNA damage-induced senescence, proliferating HF043 skin fibroblasts at low CPD were treated with etoposide (see Methods); for oncogene-induced senescence, proliferating IMR90 ER::ras cells were treated with 4-hydroxytamoxifen (4OHT) to induce *ras* expression. CTRL = control. Scale bar 50μm.

**Supplementary Figure S3. Markers of senescence.** (A, B) IL-6 SASP factor secretion is elevated in senescent versus proliferating cells. (A) Standard curve for IL-6 ELISA using purified recombinant IL-6. (B) Measurement of IL-6 in proliferating (PRO) and replicatively senescent (SEN) cells by ELISA (n=3, mean +/- SD). (C) Upregulation of p21 in senescent cells - representative western blot of p21^CDKN1^ and loading control GAPDH in proliferating (PRO) with (+) or without (-) DNA damaging agent etoposide to induce DDIS, and replicatively senescent (SEN) cells without etoposide treatment.

**Supplementary Figure S4. Senescent cells are the major donors for mitochondrial transfer.** Cells were pre-labelled with mitotracker green or mitotracker red as in main text, and co-cultured at 1:1 ratio. (A) Enlarged areas from cells in Figure 4 and 7, showing transfer of both red and green mitochondria between cells. Scale bar 50 µm. (B) Transfer of mitochondria quantified by mitotracker dye colour of donor and recipient; bars coloured by mitotracker label of recipient cells; mitotracker label of donor and recipient is indicated by colour below each column (mitotracker green shown in green and mitotracker red shown in red) P = proliferating, S = replicatively senescent. One way ANOVA, ns = not significant, *** p=0.0002. (C) Violin plot of quantification of mitochondrial donation, according to percentage of recipient cells stained with mitotracker red (R) showing donated mitotracker green foci (G) (%R cells with G mt), and percentage of recipient cells stained with mitotracker green (G) showing donated mitotracker red foci (R) (ie %G cells with R mt). Two-way ANOVA, ns= not significant, **** p<0.0001. Mean, 25th and 75th centiles, and individual data points are shown. mt = mitochondria.

**Supplementary Figure S5. Dose response curves for cells treated with (A) AZD8055 or (B) CASIN at concentrations from 0-10000nM.** Cell reducing capacity, often used as a proxy for cell viability, was assessed by alamarBlue staining. (Note 0 nM not shown as data are plotted on log scale; values are normalised to zero drug control, taken as 100%). Drug doses were tested on proliferating cells (PRO) as well as replicatively senescent (RS) and DDIS senescent cells induced by etoposide treatment (ETOP). Mean and SD are shown from n=3.

**Supplementary Figure S6: Intercellular bridge formation is sensitive to CDC42 inhibition in a dose- dependent manner.** HF043 fibroblasts at CPD 71 were incubated with 0, 1, 2 and 5 μm CASIN, an inhibitor of CDC42 for 24h then stained with FITC-WGA (green), rhodamine phalloidin (actin, red) and NucBlue Live for DNA (blue). Scale bar 100 μm.
